# National health research priorities 2024-2030: a strategic vision for comprehensive wellbeing

**DOI:** 10.17843/rpmesp.2024.414.14547

**Published:** 2024-12-05

**Authors:** Yamilée Hurtado-Roca, César Cabezas

**Affiliations:** 1 Directorate of Health Research and Innovation, National Institute of Health, Lima, Peru. Directorate of Health Research and Innovation Instituto Nacional de Salud Lima Peru; 2 Instituto Nacional de Salud, Lima, Peru. Instituto Nacional de Salud Lima Perú

The Peruvian health system faces complex challenges that require innovative solutions and comprehensive approaches. The determination of the National Lines of Health Research 2024-2030 marks a milestone by prioritizing critical issues that integrate interdisciplinary and contextual perspectives, in a collaborative effort with all actors in the research process in the country. This prioritization constitutes a roadmap to address public health problems that disproportionately impact the most vulnerable populations. Through well-delineated strategic objectives and priority areas, the country demonstrates a commitment to improving the health, equity and well-being of its population. This editorial seeks to highlight the key areas and their implications at both the national and international levels.

Research priorities should respond to the National Policy for the Development of Science, Technology and Technological Innovation (STI), given that research and innovation ecosystems should ensure that research and technological development results respond to the social, economic and environmental needs of the country; this implies that research priorities should be defined with the different actors of the STI governance system in the country, which has the priorities developed for the period 2019-2023 as a background ^1^.

Countries, particularly middle- and low-income countries, should use instruments that allow them to plan and manage research prioritization. The World Health Organization published a guideline ^2^ that interestingly addresses not only the process *per se* of identifying research priorities, but also incorporates fundamental concepts of the implementation process that are related to the constant monitoring of the dissemination of the process and of the identified priorities: execution that involves the identification of infrastructure, human resources, logistic and financial resources for its implementation and the phase of constant evaluation of the implementation process that allows for feedback and re-evaluation of strategies for its feasibility.

One of the fundamental pillars in this research framework is the approach to malnutrition and anemia in the mother-child binomial, a problem that continues to significantly affect the quality of life of the population in the first thousand days of life. This critical period, which includes pregnancy and the first three years of the child’s life, defines not only physical and cognitive development, but also vulnerability to chronic diseases throughout life. The lines of research prioritize the understanding of the causes of anemia, both nutritional and non-nutritional, and stress the importance of evaluating the diet in terms of access, bioavailability and adequacy. In addition, they propose strengthening multisectoral interventions through the development of technologies for diagnosis and monitoring, adapted to the cultural and social realities of Peru’s diverse regions. The integration of regional approaches and the consideration of social programs such as JUNTOS and CUNAMAS make these strategies particularly innovative, as they respond to the country’s diversity.

On the other hand, antimicrobial resistance is emerging as a research priority that reflects a global health problem. This challenge, which threatens to reverse decades of medical progress, requires coordinated actions under the One Health approach, integrating human, animal and environmental dimensions. In this context, it is necessary to develop innovative molecular diagnostic technologies and to evaluate strategies that promote the rational use of antimicrobials. In addition, the impact of communication campaigns and the evaluation of clinical guidelines to improve adherence are critical areas of research. Peru is in a unique position to contribute to the understanding of this phenomenon by studying the dynamics of resistance in a country with significant biodiversity and diverse antimicrobial use contexts.

In the area of mental disorders and diseases of the nervous system, research priorities highlight the need to address the social determinants that perpetuate mental health inequalities. Issues such as poverty, migration, job insecurity and environmental crises are key factors that disproportionately affect vulnerable populations. This approach seeks not only to understand the underlying causes, but also to design interventions tailored to the sociocultural context that promote prevention, early diagnosis and recovery. Mental health, often neglected by health systems, receives renewed emphasis along these lines, which could generate replicable models for other countries with similar challenges.

Non-communicable diseases (NCDs) also feature prominently in this research agenda. With a growing burden of chronic diseases such as diabetes, hypertension and obesity, Peru faces the need to integrate preventive and control strategies throughout the life course. The development of technological innovations, such as predictive tools based on artificial intelligence, could transform care in this field. National guidelines also stress the importance of multimorbidity, recognizing that NCDs rarely occur in isolation. This systemic approach may offer solutions that are not only cost-effective, but also more relevant to resource-limited contexts.

Another key issue is the impact of climate change on health, a topic of growing urgency both locally and globally. The lines of research prioritize the understanding of the relationship between extreme climate events, such as the El Niño phenomenon, and its impact on public health. In addition, mitigation and adaptation strategies are proposed that could include early warning systems and the implementation of intersectoral policies to protect the most vulnerable populations. Climate change not only directly affects health through phenomena such as heat waves or the spread of vector-borne diseases, but also exacerbates existing inequities, a central theme in these priorities.

Attention to the social determinants of health cuts across all the proposed research areas, evidencing a holistic approach. Identifying and addressing health inequalities related to socioeconomic, geographic and cultural factors is considered essential to achieve sustainable impact. The lines emphasize the need for intersectoral and multidisciplinary strategies that not only address immediate health problems, but also their structural causes.

The research agenda also includes areas of emerging relevance such as food security, tuberculosis and the implementation of digital health systems. Each of these areas is articulated with global and local challenges, seeking solutions that are innovative, cost-effective and culturally appropriate. For example, in the case of food security, interventions to promote healthy and sustainable eating habits are highlighted, as well as strategies to reduce the consumption of ultra-processed foods. In the area of digital health, the focus is on strengthening the governance of the digital ecosystem, improving the interoperability of information systems and developing emerging technologies such as artificial intelligence.

An issue that deserves special attention is the need to strengthen local research and technological development capabilities. To meet these challenges, it is essential to invest in the training of specialized human resources, foster international collaborations and ensure sustainable funding. The established priorities also call for the active participation of communities in the co-creation of solutions, recognizing that interventions must be culturally relevant and socially acceptable.

From a global perspective, Peru’s national lines of research are aligned with international trends in public health, but bring a contextual approach that enriches the debate. Antimicrobial resistance, mental disorders and the impact of climate change are topics that transcend national borders, offering opportunities for international collaborations. In addition, the emphasis on equity and cultural diversity provides a model for countries with similarly heterogeneous populations.

Peru has been using this prioritization tool since 2010, a process that has been led by the National Institute of Health, which applies criteria of knowledge gap, feasibility and impact on the general population and the health system. This process is a mechanism that seeks to prioritize resources efficiently; however, all the actors of the science, technology and innovation ecosystem have an important challenge since having a set of research priorities is only the starting point of an articulated and collaborative implementation work with the main objective of generating innovative solutions to the country’s public health problems that become challenges to be addressed through research and innovation. The Lines of Health Research for 2024-2030 have been identified ^3^ and will be formally published in the coming days.

In conclusion, the National Lines of Health Research 2024-2030 represent a significant advance in contributing to the improvement of public health in Peru. By prioritizing relevant topics, integrating multidisciplinary approaches and promoting innovative solutions, these lines establish a solid foundation to address the challenges of the 21st century. However, their success will depend on the ability to mobilize resources, coordinate intersectoral efforts and ensure a sustained commitment to the implementation of these priorities. For the international scientific community, these lines offer a unique opportunity to learn from a comprehensive and contextual approach, and to contribute to progress toward more equitable and sustainable global health.
