# Supplementary material for: National health research priorities 2024-2030: a strategic vision for comprehensive wellbeing
Source: Rev Peru Med Exp Salud Publica. 2024 Dec 5;41(4):340–2. doi: 10.17843/rpmesp.2024.414.14547 (PMC11797576; doi:10.17843/rpmesp.2024.414.14547)
Supplement: Translation

# Translation: Prioridades nacionales de investigación en salud 2024-2030: una visión estratégica para el bienestar integral

**Authors:** Yamilée Hurtado-Roca, César Cabezas

**Affiliations:** 1 Dirección de Investigación e Innovación en Salud, Instituto Nacional de Salud, Lima, Perú.; 2 Instituto Nacional de Salud, Lima, Perú.

El sistema de salud enfrenta retos complejos que requieren soluciones innovadoras y enfoques integrales. En el Perú, la determinación de las Líneas Nacionales de Investigación en Salud 2024-2030 marca un hito al priorizar temas críticos que integran perspectivas interdisciplinarias y contextuales, en un esfuerzo colaborativo con todos los actores del proceso de investigación en el país. Esta priorización constituye una hoja de ruta para abordar problemas de salud pública que impactan desproporcionadamente a las poblaciones más vulnerables. A través de objetivos estratégicos y áreas prioritarias bien delineadas, el país demuestra un compromiso con la mejora de la salud, la equidad y el bienestar de su población. Este editorial busca resaltar las áreas clave identificadas y sus implicancias tanto a nivel nacional como internacional.

Las prioridades de investigación deben responder a la Política Nacional para el Desarrollo de la Ciencia, Tecnología e Innovación Tecnológica (CTI), dado que los ecosistemas de investigación e innovación debieran procurar que los resultados de investigación y desarrollo tecnológico respondan a las necesidades sociales, económicas y ambientales del país; esto supone que las prioridades de investigación deben definirse con los diferentes actores del sistema de gobernanza de la CTI en el pais, lo cual tiene como antecedente la prioridades desarrolladas para el periodo 2019-2023 ^(^^1^.

Los paises, y en particular los de medianos y bajos ingresos, debieran utilizar instrumentos que les permitan planificar y gestionar una asignación de prioridades de investigación. La Organización Mundial de la Salud, publicó un documento orientativo^2^ que interesantemente aborda no únicamente el proceso *per se* de identificación de prioridades de investigación, sino incorpora conceptos fundamentales del proceso de implementación que están relacionados al monitoreo constante de la difusión del proceso y de las prioridades identificadas; ejecución que involucra identificación de infraestructura, recursos humanos, recursos logísticos y financieros para su implementación; y la fase de evaluación constante del proceso de implementación que permite retroalimentar ver y reevaluar estrategias para su factibilidad.

Uno de los pilares fundamentales en este marco de investigación es el abordaje de la desnutrición y la anemia en el binomio madre-niño, una problemática que sigue afectando significativamente la calidad de vida de la población en los primeros mil días de vida. Este periodo crítico, que incluye el embarazo y los primeros tres años del niño, define no solo el desarrollo físico y cognitivo, sino también la vulnerabilidad frente a enfermedades crónicas a lo largo de la vida. Las líneas de investigación priorizan el entendimiento de las causas de la anemia, tanto nutricionales como no nutricionales, y subrayan la importancia de evaluar la dieta en términos de acceso, biodisponibilidad y adecuación. Además, proponen fortalecer las intervenciones multisectoriales mediante el desarrollo de tecnologías para diagnóstico y monitoreo, adaptadas a las realidades culturales y sociales de las diversas regiones del Perú. La integración de enfoques regionales y la consideración de programas sociales como JUNTOS y CUNAMAS hacen que estas estrategias sean particularmente innovadoras, pues responden a la diversidad del país.

Por otro lado, la resistencia antimicrobiana emerge como una prioridad de investigación que refleja un problema de salud global. Este desafío, que amenaza con revertir décadas de progreso médico, exige acciones coordinadas bajo el enfoque de Una Salud, integrando las dimensiones humana, animal y ambiental. En este contexto, es necesario desarrollar tecnologías innovadoras de diagnóstico molecular y a evaluar estrategias que promuevan el uso racional de antimicrobianos. Adicionalmente, el impacto de las campañas de comunicación y la evaluación de guías clínicas para mejorar la adherencia se posicionan como áreas críticas de investigación. El Perú se encuentra en una posición única para contribuir al entendimiento de este fenómeno, al estudiar las dinámicas de resistencia en un país con una biodiversidad significativa y contextos de uso diverso de antimicrobianos.

En el ámbito de los trastornos mentales y las enfermedades del sistema nervioso, las prioridades de investigación destacan la necesidad de abordar los determinantes sociales que perpetúan desigualdades en salud mental. Problemas como la pobreza, la migración, la precariedad laboral y las crisis ambientales son factores clave que afectan desproporcionadamente a poblaciones vulnerables. Este enfoque no solo busca comprender las causas subyacentes, sino también diseñar intervenciones adaptadas al contexto sociocultural, que promuevan la prevención, el diagnóstico temprano y la recuperación. La salud mental, frecuentemente desatendida en los sistemas de salud, recibe un énfasis renovado en estas líneas, lo que podría generar modelos replicables para otros países con desafíos similares.

Las enfermedades no transmisibles (ENT) también ocupan un lugar destacado en esta agenda de investigación. Con una creciente carga de enfermedades crónicas como la diabetes, hipertensión y obesidad, el Perú enfrenta la necesidad de integrar estrategias preventivas y de control a lo largo de todo el curso de vida. El desarrollo de innovaciones tecnológicas, como herramientas de predicción basadas en inteligencia artificial, podría transformar la atención en este campo. Las líneas nacionales también subrayan la importancia de la multimorbilidad, reconociendo que las ENT rara vez ocurren en aislamiento. Este enfoque sistémico puede ofrecer soluciones que sean no solo costo-efectivas, sino también más relevantes para contextos de recursos limitados.

Otro eje clave es el impacto del cambio climático en la salud, un tema de creciente urgencia tanto a nivel local como global. Las líneas de investigación priorizan la comprensión de la relación entre eventos climáticos extremos, como el Fenómeno El Niño, y su impacto en la salud pública. Además, se proponen estrategias de mitigación y adaptación que podrían incluir sistemas de alerta temprana y la implementación de políticas intersectoriales para proteger a las poblaciones más vulnerables. El cambio climático no solo afecta directamente a la salud mediante fenómenos como las olas de calor o la propagación de enfermedades transmitidas por vectores, sino que también exacerba las inequidades existentes, un tema central en estas prioridades.

La atención a los determinantes sociales de la salud atraviesa todas las áreas de investigación propuestas, evidenciando un enfoque holístico. Identificar y abordar desigualdades en salud relacionadas con factores socioeconómicos, geográficos y culturales se considera esencial para lograr un impacto sostenible. Las líneas enfatizan la necesidad de estrategias intersectoriales y multidisciplinarias, que no solo aborden los problemas de salud inmediatos, sino también sus causas estructurales.

La agenda de investigación incluye, además, áreas de relevancia emergente como la seguridad alimentaria, la tuberculosis y la implementación de sistemas de salud digital. Cada una de estas áreas se articula con los desafíos globales y locales, buscando soluciones que sean innovadoras, costo-efectivas y culturalmente apropiadas. Por ejemplo, en el caso de la seguridad alimentaria, se destacan las intervenciones para promover hábitos alimentarios saludables y sostenibles, así como estrategias para reducir el consumo de alimentos ultraprocesados. En el ámbito de la salud digital, el enfoque está en fortalecer la gobernanza del ecosistema digital, mejorar la interoperabilidad de los sistemas de información y desarrollar tecnologías emergentes como la inteligencia artificial.

Un tema transversal que merece especial atención es la necesidad de fortalecer las capacidades locales de investigación y desarrollo tecnológico. Para enfrentar estos desafíos, es fundamental invertir en la formación de recursos humanos especializados, fomentar colaboraciones internacionales y garantizar un financiamiento sostenible. Las prioridades establecidas también abogan por la participación activa de las comunidades en la co-creación de soluciones, reconociendo que las intervenciones deben ser culturalmente pertinentes y socialmente aceptables.

Desde una perspectiva global, las líneas nacionales de investigación del Perú se alinean con las tendencias internacionales en salud pública, pero aportan un enfoque contextual que enriquece el debate. La resistencia antimicrobiana, los trastornos mentales y el impacto del cambio climático son temas que trascienden las fronteras nacionales, ofreciendo oportunidades para colaboraciones internacionales. Además, el énfasis en la equidad y la diversidad cultural proporciona un modelo para países con poblaciones igualmente heterogéneas.

El Perú viene utilizando este instrumento de priorización desde el año 2010, proceso que ha estado liderado por el Instituto Nacional de Salud, que aplica criterios de brecha del conocimiento, factibilidad e impacto en la población en general y en el sistema de salud. Este proceso es un mecanismo que busca priorizar los recursos de manera eficiente; sin embargo todos los actores del ecosistema de ciencia, tecnología e innovación, tenemos un reto importante ya que contar con un conjunto de prioridades de investigación solo constituyen el punto de partida de un trabajo de implementación articulado y colaborativo con el objetivo principal de generar soluciones innovadoras a los problemas de salud pública del pais que se convierten en desafios a ser atendidos desde la investigación e innovación. La identificación de estas Líneas de Investigación en Salud para el periodo 2024-2030 han concluido ^3^ y serán publicadas de manera formal en los próximos días.

En conclusión, las Líneas Nacionales de Investigación en Salud 2024-2030 representan un avance significativo para contribuir a la mejora de la salud pública en el Perú. Al priorizar temas relevantes, integrar enfoques multidisciplinarios y promover soluciones innovadoras, estas líneas establecen una base sólida para abordar los desafíos del siglo XXI. Sin embargo, su éxito dependerá de la capacidad de movilizar recursos, coordinar esfuerzos intersectoriales y garantizar un compromiso sostenido con la implementación de estas prioridades. Para la comunidad científica internacional, estas líneas ofrecen una oportunidad única de aprender de un enfoque integral y contextual, y de contribuir al progreso hacia una salud global más equitativa y sostenible.
